# Aerobic fitness does not modulate protein metabolism in response to increased exercise: a controlled trial

**DOI:** 10.1186/1743-7075-6-28

**Published:** 2009-06-16

**Authors:** Tracey J Smith, Matthew A Pikosky, Ann Grediagin, Carmen Castaneda-Sceppa, Lauri O Byerley, Ellen L Glickman, Andrew J Young

**Affiliations:** 1 U.S. Army Research Institute of Environmental Medicine, Natick, Massachusetts, USA; 2 Jean Mayer USDA Human Nutrition Research Center on Aging at Tufts University, Boston, Massachusetts, USA; 3 University of North Carolina at Charlotte, Charlotte, North Carolina, USA

## Abstract

**Background:**

A sudden increase in exercise and energy expenditure is associated with an increase in protein turnover and nitrogen excretion. This study examined how a sudden increase in exercise-induced energy expenditure affected whole body protein metabolism and nitrogen balance in people of differing levels of aerobic fitness. We hypothesized that alterations in whole-body protein turnover would be attenuated, and nitrogen balance would be preserved, in individual with higher levels of aerobic fitness.

**Methods:**

Eleven men, categorized as either having a lower (LOW-FIT; n = 5) or higher (FIT; n = 6) aerobic fitness level, completed a 4-d baseline period (BL) of an energy balance diet while maintaining usual physical activity level, followed by a 7-d intervention consisting of 1,000 kcal·d^-1 ^increased energy expenditure via exercise (50–65% VO_2peak_). All volunteers consumed 0.9 g protein·kg^-1^·d^-1 ^and total energy intake was adjusted to maintain energy balance throughout the 11-d study. Mean nitrogen balance (NBAL) was determined for BL, days 5–8 (EX1), and days 9–11 (EX2). Whole-body protein turnover was derived from phenylalanine and tyrosine kinetics assessed while fasting at rest on days 4, 7, and 12 using a priming dose of L-[ring-^15^N]tyrosine and a 4-h primed, continuous infusion of L-[^15^N]phenylalanine and L-[ring-^2^H_4_]tyrosine.

**Results:**

A significant main effect of time indicated that NBAL increased over the course of the intervention; however, a group-by-time interaction was not observed. Although FIT demonstrated a lower net protein oxidation and higher net protein balance compared to LOW-FIT, neither the effect of time nor a group-by-time interaction was significant for Phe flux, net protein oxidation, or derived whole-body protein synthesis and net protein balance.

**Conclusion:**

The absence of significant group-by-time interactions in protein metabolism (i.e., NBAL and whole-body protein turnover) between LOW-FIT and FIT males suggest that aerobic fitness level does not modulate protein "sparing" in response to an unaccustomed increase in energy expenditure.

## Background

Fundamental cellular, hormonal and neurological adaptations develop as sedentary individuals become physically trained. Specifically in regards to protein metabolism, studies have shown that both routine resistance [1,2] and endurance training [3-5] improve nitrogen retention. Additionally, reductions in whole-body protein turnover have been found following routine resistance training [1,2], and decreases in whole-body leucine oxidation have been documented at rest [4] and during exercise [6] following routine endurance training. In terms of muscle protein metabolism, protein synthesis increases during resting conditions in response to chronic aerobic [7,8] and resistance [9] training, and this increase is attenuated in response to chronic resistance training when measured after an acute exercise bout [9,10]. Overall, these findings suggest that fitness training imparts adaptations that down-regulate or spare the use of amino acids as an energy source and imply a more efficient use of protein by the body in fit persons.

A sudden increase in daily energy expenditure (e.g., when training volume increases) can produce an increase in protein turnover and nitrogen excretion that may be viewed as catabolic. Early work from Gontzea et al. [11], showed that men shifted from a positive to a negative nitrogen balance after beginning an endurance exercise program despite maintaining a positive energy balance, i.e. 10% more than their measured energy expenditure. Negative energy balance will further exacerbate this catabolic response [11,12]. Whether nitrogen balance and protein utilization responses to a sudden increase in aerobic exercise are modulated by fitness level is not known.

We speculated that adaptations in protein utilization associated with regular exercise could impart a protective effect against catabolism during a sudden increase in exercise energy expenditure. The research study reported herein aimed to test that hypothesis and generate new information concerning the metabolic responses of athletes who partake in rigorous pre-season training, and Soldiers who undertake intense combat missions and/or field-training exercises. If a differential response to a sudden increase in exercise with respect to protein metabolism is observed between individuals with dissimilar fitness levels, then it would be advantageous to determine if alternative nutrition requirements are warranted based on fitness level (i.e. additional protein for untrained individuals).

Therefore, the purpose of the present investigation was to examine the effect of a sudden increase in daily exercise energy expenditure while adjusting total energy intake to maintain energy balance on whole body protein turnover and nitrogen balance, and to determine if this response was modulated by variations in aerobic fitness level. Our working hypothesis was that whole-body protein turnover would increase in response to a sudden, short-term (7-d) increase in exercise energy expenditure, while daily nitrogen balance would be negative initially and rise over time as nitrogen retention improved as an adaptation to the increase in exercise. We further hypothesized that the increase in whole-body protein turnover would be attenuated, and nitrogen balance would be preserved to a greater degree, in aerobically fit individuals compared to individuals with a lower aerobic fitness level.

## Methods

### Subjects

This study was approved by the appropriate institutional review boards. Eleven healthy men gave informed, written consent to participate in this investigation following an oral and written explanation of all study procedures and risks. All subjects completed an initial screening and were medically cleared for participation in accordance with United States Army Research Institute of Environmental Medicine (USARIEM) guidelines for human use. The investigators have adhered to the policies for protection of human subjects as prescribed in Army Regulation 70-25 and USAMRMC Regulation 70-25, and the research was conducted in adherence with the provisions of 32 CFR Part 219. Investigators adhered to AR 70-25 and USAMRMC Regulation 70-25 on the use of volunteers in research.

Subjects were required to be weight stable (± 2.2 kg) for two months prior to the start of the study. Aerobically fit volunteers (FIT) were required to have a 6 month endurance training history (≤ 5 d·wk^-1^, 30 min·d^-1^), and a VO_2peak _≥ 54 or 52 ml·kg^-1^·min^-1 ^for ages 18–29 and 30–35 yrs, respectively, while low fit volunteers (LOW-FIT) were required to have an inconsistent endurance training history (< 3 d·wk^-1^, 30 min·d^-1^) and a VO_2peak _= 43 or 41 ml.kg^-1^·min^-1 ^for ages 18–29 and 30–35 y, respectively. Fitness level criteria were chosen based on the range of fitness levels that is typical in the sample population, which is U.S. Army Soldiers. Individuals who used tobacco products, had a disease or took medication known to impact macronutrient metabolism, or possessed any cardiovascular or musculoskeletal conditions that prohibited strenuous exercise were excluded.

### Experimental Design

**Figure 1:**
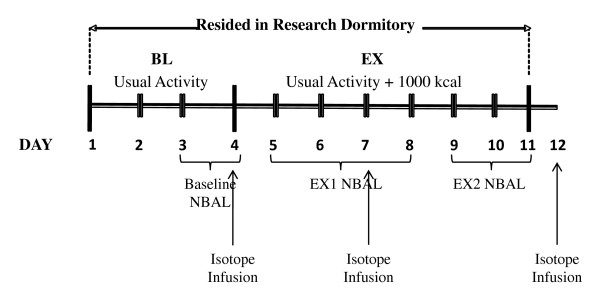
**Experimental Design**.

Figure 1 illustrates the experimental design. Volunteers resided in a research dormitory for the duration of the experimental phase of the study allowing for control and monitoring of energy intake and energy expenditure. Subjects were assigned to one of 2 groups based on their aerobic fitness level: FIT or LOW-FIT. During the first 4 days of the experimental phase energy intake and expenditure was matched to subjects' normally accustomed levels, and both groups were in energy balance. Subjects maintained diet and activity records for 3 days before beginning the experimental phase of the study to assess those normally accustomed levels. Beginning on day 5, all volunteers increased energy expenditure by 1000 kcals·d^-1 ^by completing prescribed exercise (described in 'Exercise Intervention') at intensities corresponding to 50–65% VO_2peak_. Energy intake was also increased by 1000 kcals·d^-1 ^beginning on day 5 so as to maintain energy balance. Nitrogen balance (NBAL) was assessed daily, and whole body protein turnover, derived from phenylalanine and tyrosine kinetics, was assessed in the fasted state at rest on days 4, 7, and 12, using a priming dose of L-[^15^N]phenylalanine, L-[ring-^15^N]tyrosine and L-[ring-^2^H_4_]tyrosine, and a 4-h primed, continuous infusions of L-[^15^N]phenylalanine and L-[ring-^2^H_4_]tyrosine. Baseline testing included assessment of aerobic capacity (peak oxygen uptake [VO_2peak_]), anthropometry (height and weight), body composition (DEXA), 3-d diet records, and 3-d physical activity records.

### Peak Oxygen Uptake

Peak oxygen uptake (VO_2peak_) was determined during a cycle ergometer test using indirect open circuit spirometry (True Max 2400, Parvomedics, Sandy, Utah). The exercise protocol was progressive in intensity, continuous in nature and performed indoors (20–22°C and 30–80% humidity). Volunteers first pedaled for 3 min at 100 W and then intensity was increased by 30 W every 2 min until the volunteer was unable to maintain a pedaling rate that maintained or increased O_2 _consumption.

### Anthropometry

Vertical height was measured in duplicate to the nearest 0.1 cm. Body weight was measured at baseline and twice daily (prior to morning meal, and following the evening meal) using a calibrated electronic battery-powered scale accurate to 0.1 kg. Body composition was determined by Dual Energy X-ray Absorptiometry (DEXA) (Lunar, General Electric Healthcare, Madison, WI) at baseline, and on day 11 of the intervention.

### Baseline Dietary Records

Three-day dietary records were collected from each volunteer before they began the experimental phase of the study to assess their usual energy and macronutrient intakes. All dietary records were analyzed by computer-based nutrient analysis software, Food Processor v. 8.5.0 (ESHA Research, Salem, OR). The average daily energy intake determined from these records was used in combination with the average daily energy expenditure determined from the physical activity records to establish energy intake and expenditure levels to be sustained during the first 4 days of the experimental phase of the study.

### Baseline Physical Activity Records

Three-day physical activity records were maintained by each volunteer prior to the experimental phase of the study for the purpose of achieving energy balance. Each subject's normal daily activities were grouped into different categories by duration and the corresponding metabolic equivalent (MET). The average daily energy expenditure determined from these records was used in combination with the average daily energy intake determined from the dietary records to establish the energy intake and expenditure levels to be sustained during the first 4 days of the experimental phase of the study.

### Dietary Intervention

Three to five days before starting the controlled diet, subjects were instructed to consume a diet that contained the same amount of protein as the study diet in an attempt to habituate hepatic enzymes to that specific dietary protein level. Subjects consumed a controlled diet throughout the study. The diet consisted of whole foods and liquid supplements provided to each subject in individualized amounts. All meals were prepared in the USARIEM metabolic kitchen by a Registered Dietitian. Throughout the study, the dietary protein source was consistent across groups and protein content was prescribed at 0.9 g protein·kg body weight^-1^·d^-1^. This protein level was chosen because it meets the current military dietary reference intake for protein [13]. Total energy intake for study days 1–4 was individually matched to each volunteer's energy expenditure in order to maintain energy balance. The diet was prescribed to meet the following macronutrient ranges: 8 to 13% protein, 32–37% fat, and 55% carbohydrate. Meals and snacks were served at specific times each day, but water and sugar-free non-caffeinated drinks were consumed ad libitum.

### Exercise Intervention

Usual/Normal Exercise Period: During study days 1–4, each subject's reported normal daily activities were grouped into different categories by duration and corresponding metabolic equivalent (MET) level. An exercise prescription specifying the type, intensity, and duration of all activities were tightly controlled by dividing each 24-h period into prescribed 15-min blocks at a specific MET level to duplicate the volunteers' normal daily caloric expenditure.

### Increased exercise period

During study days 5–11, volunteers increased their energy expenditure 1,000 kcal each day by adding additional exercise at 50–65% of their VO_2peak _to the original exercise prescription. For subject comfort, compliance, and to avoid injuries, exercise was distributed between various modalities (e.g., cycle ergometer, treadmill, elliptical exerciser) during each 15 min interval throughout the day. Volunteers were allowed to self select how they wanted to distribute these additional 15 minute blocks throughout the day, as long as the prescribed energy expenditure was achieved.

### Determination of Total Daily Energy Expenditure (TDEE)

Total daily energy expenditure was determined periodically throughout the study using indirect open circuit spirometry (Parvomedics, Sandy, Utah). TDEE was calculated using the following equation:



EE_sleep _was estimated via measurement of basal metabolic rate on days 1, 2, 5, and 8, EE_physical activity _was measured during each prescribed exercise activity on days 1, 5, and 8, and EE_miscellaneous _was measured during non-exercise activities (i.e., watching TV, playing video games, reading) on days 1, 5, and 8.

### Determination of Nitrogen Balance

NBAL was determined daily using the following equation:



N_in _was determined by computer-based nutrient analysis (Food Processor, v.8.5.0, ESHA Research, Salem, Oregon) of each volunteer's daily dietary intake. Total N content of daily urine, feces, and sweat collections was determined using the micro-Kjeldahl technique. Accuracy of the N_urine _was assessed using 24-h creatinine excretion. Daily fecal samples were homogenized and pooled into 3 separate collection periods according to the appearance of fecal markers (carmine red and charcoal). Total body sweat loss (N_sweat_) was estimated via a modification of the regional sweat collection method described by Lemon et al [14]. Briefly, volunteers affixed a sweat collection pad to each thigh using an adhesive dressing prior to beginning the first exercise bout of the day. These pads were removed, the urea extracted and the extract frozen for later analysis of total nitrogen following the last exercise session of the day. Total nitrogen loss was estimated by calculating the surface area of the collection pad, determining the nitrogen collected from this surface area, and extrapolating this amount to the surface area of the subject's body. Miscellaneous nitrogen losses (N_miscellaneous_, skin, hair, secretions, sweat while at rest) were estimated at 5.0 mg N/kg body weight/d [15]. Daily NBAL was calculated (g N·d^-1^) and results were then pooled and means determined for 3 separate phases, days 3–4 (baseline), days 5–8 (EX1) and days 9–11 (EX2). The division of NBAL data into three distinct phases (baseline, EX1 and EX2) was carried out in order to evaluate it against protein kinetic data, which was collected on day 4 (baseline), day 7 (EX1), and day 11 (EX2).

### Isotope-Infusion Protocol

Whole body protein turnover was assessed in the fasted state at rest on days 4, 7, and 12 using phenylalanine (Phe) and tyrosine (Tyr) kinetic models. Subjects began fasting at 1800 hours on the evening before each protein turnover assessment, and were transported to the Jean Mayer USDA Human Nutrition Research Center on Aging at Tufts University where they remained overnight in the Metabolic Research Unit under the supervision of nursing staff. Subjects were awakened the following morning at ~0450 hours. A venous catheter was inserted into an antecubital vein for isotope infusion. Another catheter was placed in the contralateral hand vein, and the hand was heated with a heating pad, for the sampling of "arterialized" blood [16]. At 0600 hours, subjects were infused with a priming dose of L-[^15^N]phenylalanine (3.9 μmol·kg^-1^), L-[ring-^2^H_4_]tyrosine (2.9 μmol·kg^-1^), and L-[ring-^15^N]tyrosine (1.4 μmol·kg^-1^), followed by a 4-hr continuous infusion (50 ml·hr^-1^) of L-[^15^N]phenylalanine (3.9 μmol·kg^-1^·h^-1^), and L-[ring-^2^H_4_]tyrosine (2.9 μmol·kg^-1^·h^-1^). "Arterialized" venous blood samples [17] were drawn immediately before the priming doses were administered, at 60-min intervals during the first 2 hours of the infusion and at 15-min intervals during the final 2 hours of the infusion for subsequent analyses of plasma isotope enrichment. Resting metabolic rate and CO_2 _production rate were measured before the priming infusions began, and for the last 5-min of each hour of the infusion. At the completion of the infusion period, catheters were removed, the subjects' arms bandaged and the volunteers were transported back to USARIEM to resume diet and exercise regimes at approximately 1200 hours.

### Calculations

Phe and Tyr kinetics were calculated using the equations described by Thompson et al. [18]. Phe and Tyr fluxes (Q; μmol·kg^-1^·h^-1^) were obtained from isotope dilution by the following equation:

(1)

where i is the rate of infusion of the tracer (μmol·kg^-1^·h^-1^), and E_i _and E_p _are the enrichments of the infusate and the plasma amino acids (Phe or Tyr) respectively. The conversion rate of Phe to Tyr (Q_pt_; μmol·kg^-1^·h^-1^) was calculated from the following equation:

(2)

where Q_t _and Q_p _are the flux rates for Tyr and Phe estimated independently by the primed constant infusions of [^2^H_4_] Tyr and [^15^N] Phe respectively, E_p _and E_t _are the respective enrichments of [^15^N] Phe and [^15^N] Tyr in plasma, and i_p _is the infusion rate of [^15^N] Phe.

### Whole Body Protein Turnover

Whole body protein kinetics in the fasted steady state were estimated from Phe and Tyr kinetics based on the following equation:

(3)

where S_p _is the loss of Phe from the free amino acid pool to protein synthesis, and B_p _is the rate of entry of Phe into the free amino acid pool from protein breakdown. Because Phe is not synthesized endogenously, its flux in the fasted state is an index of whole-body protein breakdown because it represents Phe derived exclusively from whole-body proteolysis. Similarly, the conversion rate of Phe to Tyr or Phe hydroxylation can serve as an index of net protein oxidation. Assuming that most Phe not oxidized is used for protein synthesis, the disposal rate of non-oxidative Phe, derived by subtracting the conversion rate of Phe to Tyr from Phe flux can be considered an index of whole-body protein synthesis. Lastly, net protein balance can be calculated by subtracting Phe flux (or whole body protein breakdown) from whole-body protein synthesis.

### Statistical Analysis

Data from Gaine et al [4] was used to determine sample size. Five subjects per group are necessary to detect a 3.0 umol·kgFFM^-1^·h^-1 ^difference in net protein oxidation between FIT and LOW-FIT (from BL to EX2) with a standard deviation of 1.5 umol·kgFFM^-1^·h^-1^, alpha equal to 0.05 and power at 0.8. Results are presented as mean (± SD). Statistical analysis was completed using the SPSS statistical package version 15.0 (SPSS Inc., Chicago, IL). Students T-test was utilized to compare pre- and post-study anthropometric data (i.e., body weight, body fat, and fat-free mass), while repeated measures ANOVA, with group as the between subjects factor, was employed to determine differences in NBAL and whole-body protein turnover. Significant main or interaction effects were analyzed using Tukey's post hoc test. A "p" value of ≤ 0.05 was considered statistically significant.

## Results

### Subject Characteristics

Groups did not differ with respect to age, height or weight, but did differ with respect to VO_2peak_, body fat and fat-free mass. Descriptive characteristics for the 2 experimental groups are presented in Table 1.

**Table 1:** Baseline Subject Characteristics

	LOW-FIT (n = 5)	FIT (n = 6)
Age (y)	23 ± 5^a^	24 ± 2^a^
Height (cm)	173 ± 6^a^	177 ± 6^a^
Weight (kg)	74 ± 9^a^	76 ± 3^a^
Body Fat (%)	19 ± 3^a^	16 ± 3^b^
Fat Free Mass (kg)	59.9 ± 5.2^a^	63.7 ± 4.7^b^
*VO*_2*peak *_(ml·kg^-1^·min^-1^)	38 ± 3^a^	57 ± 8^b^
Protein intake (g·kg FFM^-1^·d^-1^)	1.3 ± 0.4^a^	1.8 ± 0.4^b^

^a, b ^Means not sharing a superscript indicate difference (p < 0.05)

### Dietary Intervention

Protein intake in both groups was slightly higher than the prescribed 0.9 g·kg^-1^·d^-1 ^(~1.0 ± 0.0 g·kg^-1^·d^-1^). Actual dietary intakes during the study are presented in Table 2. When protein intake was analyzed as g·kg FFM^-1^·d^-1^, FIT consumed less protein compared to LOW-FIT during the baseline period (0.07 ± 0.04 g·kg FFM^-1^·d^-1^, P = 0.04) and during the exercise period (0.06 ± 0.04 g·kg FFM^-1^·d^-1^, P = 0.04).

**Table 2:** Actual Dietary Intakes

	LOW-FIT (n = 5)		FIT (n = 6)	
	BL	EX	BL	EX

Energy Intake (kcal·d^-1^)	2936 ± 394^a^	3980 ± 373^a^	3767 ± 700^b^	4809 ± 612^b^
Protein (g·kg FFM^-1^·d^-1^)	1.24 ± 0.04^a^	1.22 ± 0.04^a^	1.17 ± 0.05^b^	1.16 ± 0.04^b^
Protein (% total intake)	10.2 ± 1.3	7.3 ± 0.7	8.1 ± 1.2	6.2 ± 0.7
CHO (% total intake)	53.8 ± 1.1	56.1 ± 1.8	54.5 ± 1.1	55.8 ± 0.9
Fat (% total intake)	37.1 ± 1.1	37.5 ± 0.7	38.6 ± 1.8	39.0 ± 1.6

^a, b ^means not sharing a superscript indicate between group differences during BL and EX (P < 0.05); FFM = fat-free mass

### Body Composition

As expected, no significant changes in body weight or body composition were observed over the 11-d period, as energy balance was maintained in both groups.

### Nitrogen Balance

**Figure 2:**
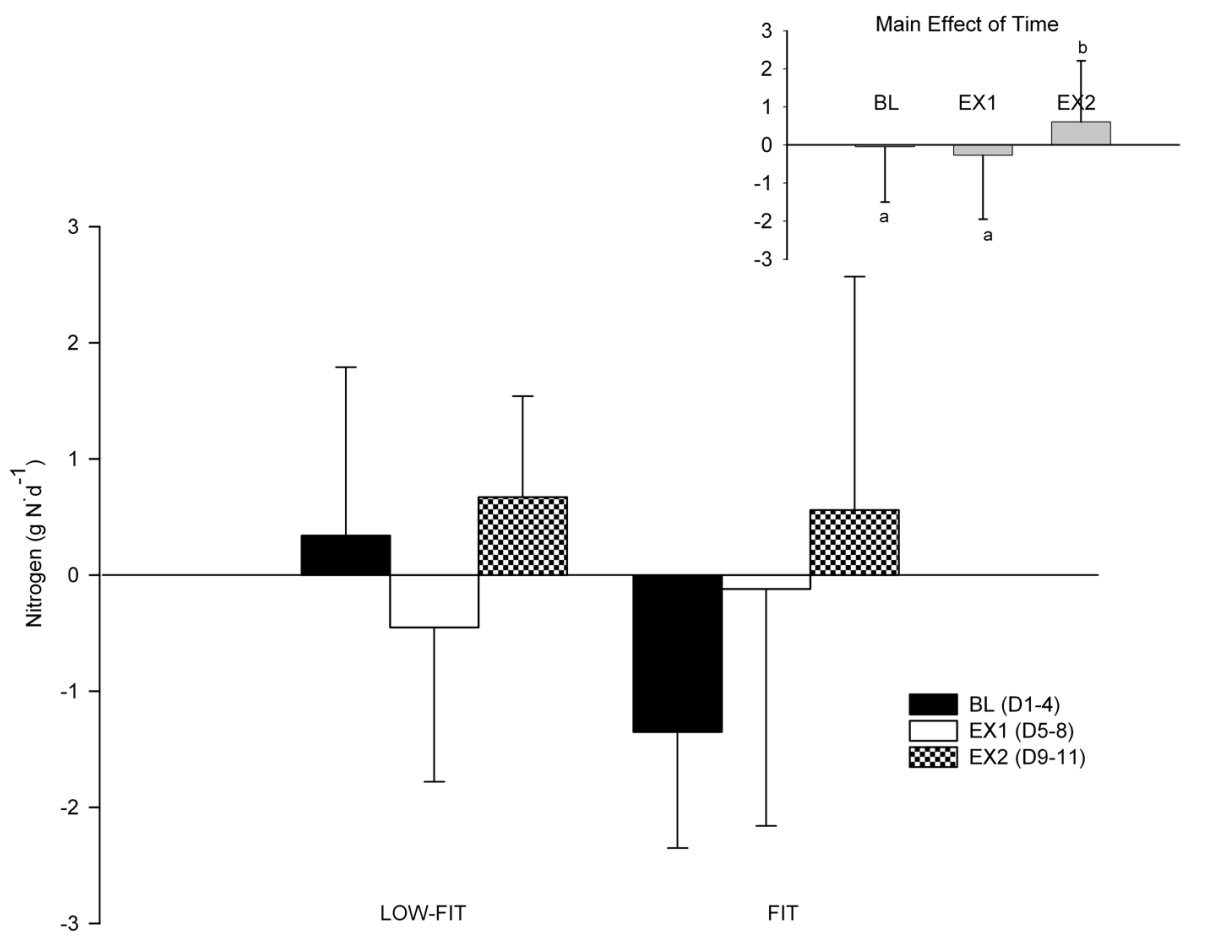
**Nitrogen balance (gN·d^-1^) for LOW-FIT and FIT at baseline (D3–4), EX 1 (D5–8), and EX 2 (D9–11)**. Inset displays the main effect of time.

Pooled NBAL data from study days 3–4 (BL), 5–8 (EX 1) and 9–11 (EX 2) did not reveal a group-by-time interaction (p > 0.05) (Figure 2). A significant main effect of time (Figure 2 inset) indicated that NBAL increased from baseline (-0.51 ± 1.45 g N) and D5–8 (-0.27 ± 1.68 g N) to the end of the intervention (D9–11: 0.61 ± 1.60 g N), P = 0.04. Further NBAL did not significantly differ from zero for either group.

### Phenylalanine and Tyrosine Kinetics

Steady state was achieved by ensuring that plasma enrichment of [ring-^15^N]tyrosine, [ring-^2^H_4_]tyrosine, and L-[^15^N]phenylalanine did not fluctuate more than 10% from the mean for each volunteer over the course of the infusion period. In cases where enrichment values varied greater than 10%, those values were excluded from the analysis. Phe and Tyr kinetics are corrected for fat free body mass, which is supported by their significant correlation with FFM and the fact that the intercepts were not significantly different from zero. Neither the effect of time nor a group-by-time interaction was significant for whole-body protein breakdown (i.e., Phe flux or Q_p_,), net protein oxidation (i.e., conversion rate of Phe to Tyr or Q_pt_), derived whole-body protein synthesis (Q_p _- Q_pt_), or derived net protein balance [(Q_p _- Q_pt_) - Q_p_]. A significant main effect was observed between groups, indicating that FIT experienced a lower net protein oxidation (P = 0.02), and higher net protein balance (P = 0.02), compared to LOW-FIT. Additionally, there was a tendency for higher protein synthesis for FIT (49.9 ± 4.0 μmol·kg FFM^-1^·h^-1^) compared to LOW-FIT (43.5 ± 4.4 μmol·kg FFM^-1^·h^-1^) (p = 0.06). Data for Phe and Tyr kinetics are presented in Table 3.

**Table 3:** Phenyalanine and Tyrosine Kinetics

	LOW-FIT (n = 5)				FIT (n = 6)		
	BL (D4)	EX1 (D7)	EX2 (D11)	BL (D4)	EX1 (D7)	EX2 (D11)	P-values (Group × Time Effect)

Q_p_ (umol·kgFFM^-1^·h^-1^)^a^	51.6 ± 7.0	48.7 ± 3.1	50.0 ± 6.2	55.2 ± 7.1	55.4 ± 3.9	54.7 ± 8.0	0.73
Q_pt_ (umol·kgFFM^-1^·h^-1^)^b^	7.6 ± 1.9	6.2 ± 1.6	5.9 ± 1.7	6.0 ± 2.1	4.9 ± 2.6	4.8 ± 1.4	0.97
Q_p _- Q_pt_ (umol·kgFFM^-1^·h^-1^)^c^	44.1 ± 6.3	42.4 ± 2.6	44.1 ± 5.7	49.3 ± 6.1	50.5 ± 4.3	49.9 ± 7.6	0.62
(Q_p _- Q_pt_) - Q_p_ (umol·kgFFM^-1^·h^-1^)^d^	-7.5 ± 1.9	-6.3 ± 1.6	-5.9 ± 1.7	-6.0 ± 2.1	-4.9 ± 2.6	-4.8 ± 1.4	0.29

^a ^whole-body protein breakdown

^b ^net protein oxidation (conversion rate from phenylalanine to tyrosine)

^c ^whole-body protein synthesis

^d ^net protein balance

Repeated measures ANOVA with aerobic fitness level (i.e. VO_2peak_) as a covariate was also conducted to elucidate whether the range of VO_2peak _between subjects was large enough to influence measures of whole-body protein turnover, and, if so, did VO_2peak _significantly influence measures of whole-body protein turnover. Results indicated that the range of VO_2peak _was not large enough between subjects to influence whole-body protein breakdown (i.e., Phe flux or Q_p-_,) or derived whole-body protein synthesis (Q_p _- Q_pt_), but was large enough to influence net protein oxidation (i.e., conversion rate of Phe to Tyr or Q_pt_) and derived net protein balance [(Q_p _- Q_pt_) - Q_p_], p < 0.05. Nevertheless, there was no significant interaction between aerobic fitness level and time for these markers of whole body protein turnover.

## Discussion

This investigation examined the impact of a 1000 kcals·d^-1 ^increase in exercise energy expenditure on NBAL and whole-body protein turnover in healthy male volunteers who differed in aerobic fitness level. The main objective of the study was to determine whether the effects of increased exercise on protein metabolism would be modulated by fitness level. We hypothesized that whole-body protein turnover would increase in both fit and unfit individuals in response to the increase in exercise energy expenditure, while daily NBAL would be negative initially and rise over time as nitrogen retention improved with adaptation to the increased exercise. We also thought that the increase in whole-body protein turnover would be attenuated, and NBAL would be preserved to a greater degree in FIT than LOW-FIT persons.

Contrary to our hypothesis, we found no significant changes in whole-body protein turnover in response to the increased exercise energy expenditure (i.e., no effect of time or group-by-time interaction). Further, when the whole-body protein turnover data for both groups was combined and analyzed using aerobic fitness level (VO_2peak_) as a covariate, no significant associations between VO_2peak _and markers of whole body protein turnover were observed. We did, however, observe a significant group effect as LOW-FIT had higher levels of net protein oxidation compared to FIT and FIT had higher levels of net balance compared to LOW-FIT. Additionally, we demonstrated similar changes (time-course and magnitude) in NBAL due to increased energy expenditure for FIT and LOW-FIT: NBAL was initially negative in response to the exercise intervention, but then increased as volunteers adapted to the additional exercise. Contrary to our hypothesis, however, this observation was not dependent on aerobic fitness level.

The NBAL response we observed is consistent with previous reports by Gontzea et al. [11] who demonstrated that when sedentary subjects initiated an exercise regime, and caloric intake was 10% greater than expenditure, NBAL went from positive to negative, but later returned to zero by day 15. In our investigation, FIT and LOW-FIT achieved neutral and positive NBAL, respectively, after only 7 days of exercise training, indicating an adaptation in protein metabolism to the increased exercise and that protein intake was sufficient when subjects maintained energy balance. Indeed, although percent of energy contributed from protein was slightly below the lower level of the Acceptable Macronutrient Distribution Range [19], for both groups, protein intake calculated per kg body weight per day was adequate according to current recommendations [19].

Contrary to our expectations, we observed a negative nitrogen balance in the FIT group during the baseline period. It is possible that the 3- to 5-d adaptation period used in this investigation may not have been of sufficient duration to achieve a true adaptation to the protein level provided during the study for the FIT volunteers who habitually consumed ~1.6 g of protein·kg^-1^·d^-1^, which we previously acknowledged [20]. Although the major initial changes in nitrogen excretion occur within approximately 5–7 days in adults according to a World Health Organization (1985) report [21], others have suggested a longer adaptation period may be necessary depending on the discrepancy between the habitual and "new" protein intake [22]. Additionally, the level of dietary control during the adaptation period could also have impacted our baseline nitrogen balance values. Volunteers were still free-living during this adaptation period, and food was not provided, therefore, it is possible that they may not have adhered to the run-in diet closely enough.

Despite the initial negative nitrogen balance observed in the FIT group, volunteers still achieved NBAL after 7 days of exercise training and our NBAL results are consistent with reports by Butterfield and Calloway [3] and Todd et al. [5], showing that adequate energy intake maintained NBAL in the face of an unaccustomed increase in exercise energy expenditure. Indeed, we previously reported that fit volunteers consuming 1.0 g protein·kg^-1^·d^-1^, who did not match their energy intake with this increased energy expenditure were in a state of negative NBAL throughout this 7-d period [23].

Our results demonstrate a significant between group effect for whole-body protein turnover, regardless of time. The fact that we observed a higher net protein oxidation in LOW-FIT compared to FIT independent of time might have been expected, given the decrease in protein oxidation that was observed in response to endurance training both at rest [4] and during exercise [6]. Our results demonstrating a less negative net balance for FIT compared to LOW-FIT independent of time is also not surprising, given that trained persons are more efficient at protein metabolism [3-5]. We also observed a tendency (P = 0.06) for higher whole-body protein synthesis in FIT compared to LOW-FIT independent of time, and that has not been reported before. Observed differences in net protein balance between the NBAL and tracer methodology (that is, net balance remained negative whereas NBAL shifted to neutral or positive) are likely due to the fact that tracer measures were performed in the fasted state while NBAL included fasted and fed periods. Although measurements of whole body protein metabolism may not be reflective of changes in fractional synthetic rate at the muscle level, this may provide insight into our findings since fractional synthetic rate in the muscle has been reported to increase in response to chronic aerobic [7,8] and resistance exercise training [9].

Whole-body protein turnover responses to an acute (1–7 days) increase in exercise energy expenditure of the magnitude employed in this study (1000 kcals·d^-1^) has not been investigated in either sedentary or aerobically fit individuals. Contrary to our hypothesis, our whole-body protein turnover results do not demonstrate a significant time effect or group-by-time interaction, in response to an increase in aerobic energy expenditure. One possible explanation for why differences were not observed may be related to different protein intakes between groups. While protein intake was matched for grams·kg body weight^-1^·day^-1^, differences in FFM between the groups resulted in a statistically significant difference in grams of protein·kg FFM^-1^·day^-1 ^(i.e. protein intake was higher for LOW-FIT vs. FIT). However, we contend that it is unlikely that this difference between groups (< 1.0 g protein per day) would elicit a true physiological response and confound our findings. However, the reality that FIT had to adapt to the "new" protein intake, in addition to the increase in exercise energy expenditure, may have masked any group-by-time differences in whole-body protein turnover responses.

It is probably inappropriate to compare our results to studies employing chronic aerobic training, since those studies sought to elucidate "training adaptations" in regards to whole-body protein turnover. We did not expect to elicit adaptations over the course of 7 days, but sought to determine if aerobic fitness level exerted a protective effect on protein utilization and protein balance when individuals were in a catabolic state. In any event, the changes reportedly occurring in response to experimental aerobic training programs are inconsistent, with some reporting no change in resting leucine oxidation [6,8] and another suggesting a decrease in leucine oxidation [4].

In terms of the tracer measurements, McKenzie et al [6] observed an improvement in protein utilization when leucine oxidation was measured during exercise, but no change in protein utilization when leucine oxidation was measured at rest in response to 38 days of endurance training. Therefore, we may have detected group-by-time interactions if we had measured protein utilization during, or immediately after exercise, instead of at rest. Additionally, the use of multiple tracers to more accurately assess whole-body protein turnover, particularly the addition of a branched-chain amino acid tracer such as leucine, may have yielded differential results since the metabolism of one particular amino acid may not be representative of all the amino acids in the body [24]. We chose phenylalanine as a tracer because it is not synthesized endogenously or oxidized by muscle. Additionally, results derived from this method have been shown to be similar to those from the leucine method [18]. This approach is not without its weaknesses [25,26], however, which we previously acknowledged [27].

The fitness level of our untrained volunteers is perhaps another reason why we did not detect differences in whole-body protein turnover between trained and untrained volunteers in response to the increased exercise. Our population of low-fit volunteers routinely expended ~3000 kcals·d^-1 ^and increased to ~4000 kcals·d^-1 ^during the intervention. Therefore, they perhaps should be characterized as "moderately active". This is in contrast to the 'sedentary' volunteers utilized in other acute exercise trials similar to ours who regularly expended an average of ~2000 kcals·d^-1 ^during baseline testing and ~3000 kcals·d^-1 ^during the exercise intervention [11]. Therefore, our volunteers do not fit the traditional definition of "sedentary", and perhaps the moderate activity demonstrated by our "low-fit" volunteers is enough to elicit "training adaptations" that spare body nitrogen and attenuate protein utilization. In order to further elucidate this notion, we combined the data from both groups and conducted repeated measures ANOVA with aerobic fitness level (VO_2peak_) as a covariate. Indeed, for whole-body protein synthesis and derived whole-body protein breakdown, the range in VO_2peak _was not large enough between volunteers to significantly influence these outcome measures. However, when the range in VO_2peak _was large enough to influence net protein oxidation and derived net balance, no significant associations were observed.

Despite the potential limitations presented herein, this rigorously controlled study provides insight into the notion that aerobic fitness level modulates the effects of increased exercise on protein metabolism. Similar to other investigations, our findings demonstrate that aerobic fitness level modulates whole-body protein turnover at rest regardless of the unaccustomed increase in exercise. However, our results did not reveal any significant changes in resting measures of whole-body protein turnover between fit and low-fit males in response to the increased exercise, nor was there any substantial evidence that aerobic fitness level influences net protein oxidation or derived net balance in response to increased exercise. These findings suggest that chronic aerobic training does not impart metabolic adaptation to spare body protein in response to an unaccustomed increase in energy expenditure when measured at rest.

## Conclusion

Regardless of fitness level, a protective adaptation to an unaccustomed increase in physical activity appears to develop fairly rapidly (i.e., 7 days or less), thereby mitigating amino acid oxidation, at least when conditions allow energy balance to be maintained. Additionally, protein metabolism homeostasis was achieved with energy sufficiency without increasing protein intake. It would be advantageous to assess protein utilization immediately after an acute exercise bout to determine the effect of an unaccustomed increase in energy expenditure between trained versus untrained volunteers. Further research is also warranted to assess protein utilization in trained versus untrained volunteers in response to an exercise-induced energy deficit, to confirm or refute the null hypothesis we demonstrated herein. Additionally, since measurements of whole-body protein turnover may not be reflective of changes in rates of skeletal muscle protein synthesis [28-31], future studies should address newer approaches that will directly measure skeletal muscle protein turnover.

## Disclaimers

The opinions or assertions contained herein are the private views of the author(s) and are not to be construed as official or reflecting the views of the Army or the Department of Defense.

Any citations of commercial organizations and trade names in this report do not constitute an official Department of the Army endorsement of approval of the products or services of these organizations.

## Competing interests

The authors declare that they have no competing interests.

## Authors' contributions

TS coordinated data acquisition, performed the statistical analysis, and drafted the manuscript. MP participated in data acquisition and analysis, and helped to draft the manuscript. AG conceived the study, and participated in its design and coordination. CC participated in study design and was responsible for data acquisition related to phenylalanine and tyrosine kinetics. LB was responsible for analysis of phenylalanine and tyrosine kinetics. EG participated in data acquisition and analysis related to the exercise intervention. AY made substantial contributions to study conception and design and critically revised the manuscript for intellectual content. All authors read and approved the final manuscript.
